# Identification and Characterization of microRNAS from *Entamoeba histolytica* HM1-IMSS

**DOI:** 10.1371/journal.pone.0068202

**Published:** 2013-07-12

**Authors:** Fermín Mar-Aguilar, Victor Trevino, Jannet E. Salinas-Hernández, Marcela M. Taméz-Guerrero, María P. Barrón-González, Eufemia Morales-Rubio, Jaime Treviño-Neávez, Jorge A. Verduzco-Martínez, Mario R. Morales-Vallarta, Diana Reséndez-Pérez

**Affiliations:** 1 Departamento de Biología Celular y Genética, Facultad de Ciencias Biológicas, Universidad Autónoma de Nuevo León, San Nicolás de los Garza, Nuevo León, México; 2 Departamento de Ingeniería Biomédica, Instituto Tecnológico y de Estudios Superiores de Monterrey, Monterrey, Nuevo León, México; University of Medicine and Dentistry of New Jersey, United States of America

## Abstract

**Background:**

*Entamoeba histolytica* is the causative agent of amebiasis, a disease that is a major source of morbidity and mortality in the developing world. MicroRNAs (miRNAs) are a large group of non-coding RNAs that play important roles in regulating gene expression and protein translation in animals. Genome-wide identification of miRNAs is a critical step to facilitating our understanding of genome organization, genome biology, evolution, and post-transcriptional regulation.

**Methodology/Principal Findings:**

We sequenced a small RNA library prepared from a culture of trophozoites of *Entamoeba histolytica* Strain HM1-IMSS using a deep DNA sequencing approach. Deep sequencing yielded 16 million high-quality short sequence reads containing a total of 5 million non-redundant sequence reads. Based on a bioinformatics pipeline, we found that only 0.5% of these non-redundant small RNA reads were a perfect match with the drafted *E. histolytica* genome. We did not find miRNA homologs in plant or animal miRNAs. We discovered 199 new potential *Entamoeba histolytica* miRNAs. The expression and sequence of these Ehi-miRNAs were further validated through microarray by µParaflo Microfluidic Biochip Technology. Ten potential miRNAs were additionally confirmed by real time RT-PCR analysis. Prediction of target genes matched 32 known genes and 34 hypothetical genes.

**Conclusions/Significance:**

These results show that there is a number of regulatory miRNAs in *Entamoeba histolytica*. The collection of miRNAs in this parasite could be used as a new platform to study genomic structure, gene regulation and networks, development, and host-parasite interactions.

## Introduction

*Entamoeba histolytica* causes amebic dysentery and liver abscesses. Most infections with *E. histolytica* are asymptomatic and only one in five infections leads to disease [1], [2], [3]. The parasite and host factors that control the outcome of this infection (asymptomatic infection versus amebic dysentery and/or liver abscesses) are not well understood, although there is emerging evidence that host, parasite and environmental factors influence the outcome of infection [1], [2], [3]. Alteration in the transcription of certain crucial genes is also likely to contribute to the outcome of infection. The latent period between infection and disease in humans suggests that the parasite adapts to the host via altered gene expression [4]. This is best illustrated by the ability of *E. histolytica* to select for increased virulence of an axenic strain of *E. histolytica* by multiple rounds of passage through animals [5].

MicroRNAs (miRNAs) are part of the interference RNA (iRNA) machinery for post-transcriptional regulation. miRNAS are 21–24 nucleotide-long RNA fragments that repress mRNA by partially binding to target mRNAs by base-pairing. Because a large fraction of protein-coding genes are under miRNA control, production of the appropriate level of specific miRNAs at the right time and place is integral to most gene regulation pathways [6].

The RNAi machinery seems to be functional in *Entamoeba* but it appears to be different compared with the RNAi machinery in other model systems. Most components of typical RNAi pathway genes are present in the *E. histolytica* genome. For example the AGO gene (*EHI_125650*), part of the RNAi pathway, has been proven to be functional *in vivo*. Nevertheless, Dicer, a key enzyme that cleaves un-mature miRNAs for activation is still elusive in *E. histolytica*. In contrast, small RNA cloning efforts have revealed the existence of a complex population of small RNAs that are likely to be involved in regulating gene expression. Interestingly, since the 5′-polyP small RNAs are Dicer-independent, it is possible that *E. histolytica* could have evolved some unknown mechanism to manipulate gene expression without a Dicer enzyme. The discovery of an abundant population of 27-nucleotide-long small iRNAs (siRNA) with an unusual 5′-polyP structure suggests that the siRNA pathway is functional in *E. histolytica* [7], [8]. This implies that *E. histolytica* can generate miRNAs that play important roles in post- transcriptional regulation.

Some putative miRNAs from *E. histolytica* were predicted using a bioinformatic approach [9]. However, experimental identification of small RNA molecules may increase our knowledge of microRNAs, reveal unique classes of riboregulators [10], and develop novel biomarkers for this parasitic disease [11]. Therefore, the goal of this study was to identify putative miRNA from an isolate of small RNA molecules from *E. histolytica*. We used throughput Solexa technology to sequence the *E. histolytica* small RNA library. The sequencing data were further analyzed and filtered for miRNA criteria. The miRNAs described here add to the growing database of novel miRNAs.

## Results

### Overview of the Sequencing Results

**Figure 1:**
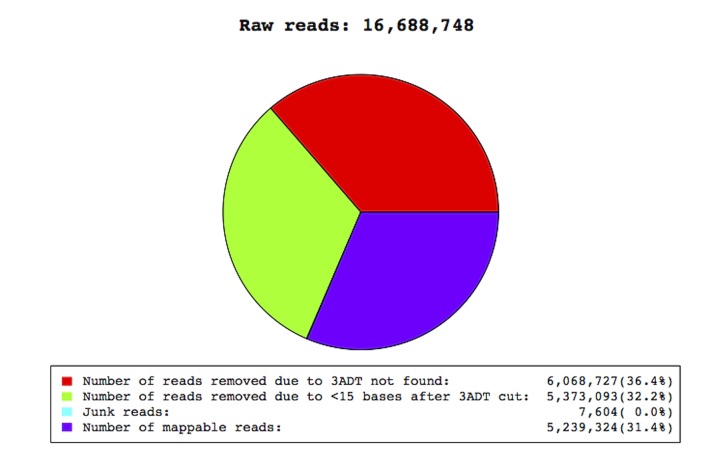
Pie plot of data filtering and database mapping. Distribution of reads obtained by deep Sequencing of trophozoites from Entamoeba histolytica.

An Illumina GAIIx high throughput sequencing generated 16,688,748 individual sequencing reads with satisfactory base quality scores for trophozoites of *Entamoeba histolytica* strain HM1-IMSS. After removing the sequencing adaptor, artificial sequences, or unresolved nucleotides, we generated 5,239,324 mappable sequences, as shown in Figure 1. We performed several “mappings” on unique seqs against pre-miRNA and mature miRNA sequences listed in the latest release of miRBase [12], or genome based on the public releases of appropriate species. The filtered unique seqs were aligned against *pre-miRNAs* of *E. histolytica* specified in miRbase. The mapped unique seqs were grouped as “unique seqs mapped to selected *pre-miRNA* in miRbase”, while the remaining ones were grouped as “unique seqs un-mapped to selected *pre-miRNA* in miRbase”. Unique seqs were divided in 4 groups; the first 3 groups have sequences of pre-miRNA mapped to miRbase, but not mapped to *E. histolytica* genome, while group 4 contains unique seqs not mapped to miRbase. Group 4 was further divided depending on potential to form hairpins (group 4a), or inability to form hairpins (group 4b). A flowchart of the data analysis and number of mappable reads for each group is shown in Figures S1 and S2, respectively.

**Figure 2:**
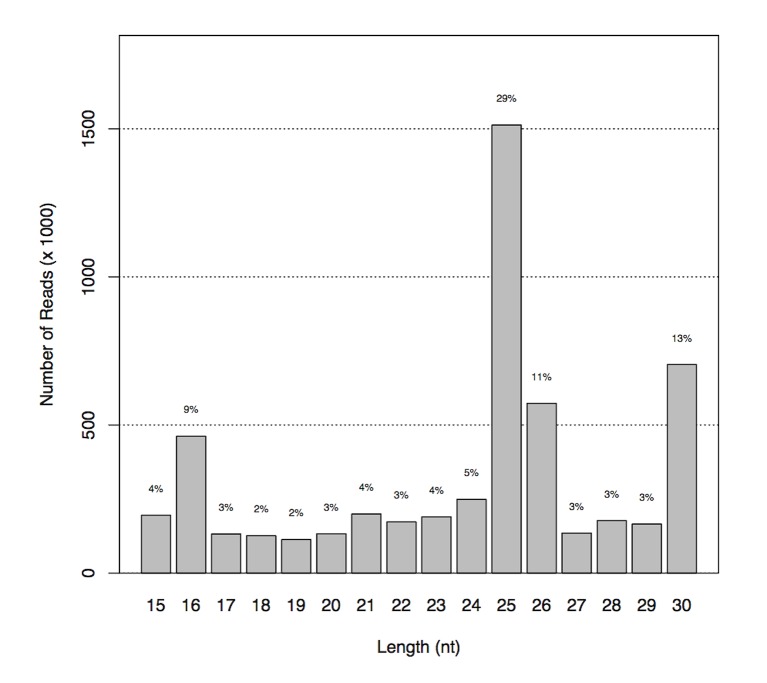
Length distribution of mappable reads. Most of the reads that are likely to be miRNAs are of 25 to 26 nucleotides in size accounting for 40% of the total mappable reads.

The relative abundance of these mappable reads by RNA size (15–30 nt) is shown in Figure 2. Most of the reads that are likely to be miRNAs are of 25 to 26 nucleotides in size, accounting for 40% of the total mappable reads.

### Identification of Novel miRNAs in Trophozoites of *Entamoeba histolytica*

To further identify potential *E. histolytica* miRNAs, we considered properties that have proved useful for distinguishing bilaterian miRNAs from other types of small RNAs (see Materials and Methods section).

We aligned the small RNAs (5,239,324 total reads) to known animal miRNA precursors to identify conserved miRNAs. We obtained 1,706 (0.03%) sequences mapped to miRbase (pre-miRNAs) but these cannot form hairpins (group 2b in Figure S1). We did not observe any sequence mapped to other genomes (group 1a in Figure S1). Interestingly we counted 96,880 reads (78,754 for group 3a and 18,126 for 3b) that contain sequences that map to mature miRNA or pre-miRNA in miRbase but not map to *E. histolytica* genome. These sequences may be caused by the presence of bacteria in the vacuoles of *E. histolytica* and therefore were discarded.

Most of the reads not mapped to miRbase mapped to *E. histolytica* genome but cannot form hairpins (61.2%, group 4b in Figures S1 and S2) or do not represent RNA families, mRNAs, or repetitive elements (24.4%, “Others” in Figures S1 and S2) Twelve percent were not mapped to *E. histolytica* (nohit in Figures S1 and S2). The remaining reads, 27,444 or 0.5% of all mapped reads not mapped to miRbase, potentially form hairpins and therefore may be strong miRNAs candidates in *Entamoeba histolytica*.

After filtering reads, the analysis revealed 199 miRNAs that were recognized in *E. histolytica* (Table S2). *E. histolytica* miRNA precursors are 58–142 nucleotides (nt) long and most showed the typical RNA hairpin structure, as denoted in Additional File 1. The length of these novel miRNAs ranged from 15 to 30 nt, and the minimum free energy (MFE) of the novel miRNAs varied from −65.70 to −7.19 kcal mol^−1^. The average MFE of novel miRNAs was −21.65 kcal mol^−1^, which is lower than the MFE of tRNA and rRNA. This result was similar to those of Bonnet et al. [13] indicating that the majority of miRNAs exhibit a folding free energy that is lower than the MFE of shuffled sequences.

Other authors have obtained miRNA sequences by bioinformatic approaches [9]. To date, these predictions have not been confirmed experimentally.

We compared the 199 putative miRNA sequences we obtained with the 32 sequences (grouped in 17 miRNAs) proposed by De et al. [9] using blast. We found only one 12-nt hit in the Ehi-miR-141 sequence (27 nt long) that matches the sequence of Eh-mir-17 predicted by De et al. We also compared the surrounding putative genomic regions (139 nt in average) and found 8 hits; the largest match was 13 nt. These results suggest that our putative miRNAs sequences are not similar to those proposed by De et al. [9].

### Target Gene Prediction for miRNAs in *Entamoeba histolytica*

Prediction of target genes is a complex process and requires further experimentation to estimate the false-discovery rate. We used miRanda, an open-source computational algorithm with a known false-discovery rate [14], [15]. There are 8201 protein-coding genes annotated for *E. histolytica,* and 90% of them do not have an annotated 3′ UTR. Moreover, the annotated genes have 3′ UTR sequences with variable length. Therefore we used the 340 genes with annotated 3′ UTR to estimate the expected maximum length of their 3′ UTR region by the mean plus two standard deviations (observed mean = 140 and sd = 180). Thus, we extracted −30 to +500 nucleotides relative to the stop codon. Wei et al. [16] set the parameter of minimum free energy (MFE) in −14 kCal mol^−1^ but other authors, such as Huang et al., [17] set MFE in −25 kCal mol^−1^. For this research, we choose an intermediate value of MEF (−18 kCal mol^−1^) to perform the analysis with miRanda Algorithm [14]. We obtained 66 putative target genes; 32 are hypothetical and 34 genes have known functions (Table S1). These results suggest that miRNAs in *E. histolytica* could regulate 1 to 8 different targets including transcription factors.

### Microarray Validation of Putative *E. histolytica* miRNAs

**Figure 3:**
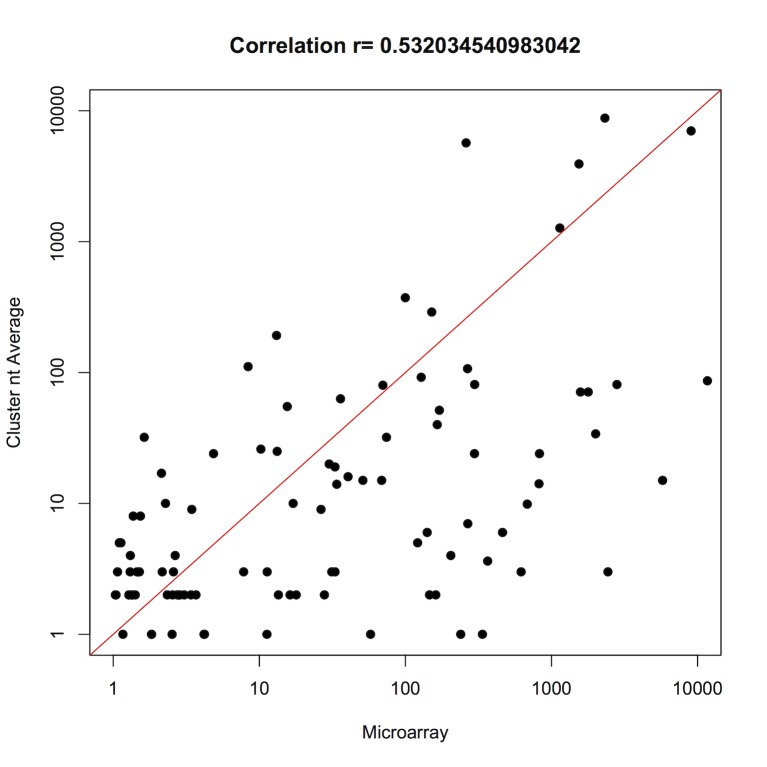
Correlation of cluster nt average vs microarray. The correlation of 0.532 suggesting agreement between expression estimations from the microarray and deep sequencing.

To confirm the existence of the newly identified *E. histolytica* miRNA candidates, we hybridized the same RNA preparation used in deep sequencing with µParaflo Microfluidic Biochip Technology [18]. Probes were based on the 199 candidate miRNA sequences obtained in this work. We used microarray spots and sequencing counts greater than 1 to avoid comparisons of poorly hybridized spots and unique sequences that could be caused by non-biological effects. Figure 3 shows an acceptable correlation of 0.532, suggesting agreement between expression estimates from the microarray and deep sequencing.

### Real Time PCR Validation of Putative *E. histolytica* miRNAs

Some miRNAs were amplified by RT-PCR to further validate the presence of candidate miRNAs of *E. histolytica* identified from the sequencing results. We selected 10 candidate miRNAs from sequencing data across a wide range of copy levels (Ehi-miR-2, Ehi-miR-5, Ehi-miR-8, Eh-miR-12, Ehi-miR-13, Ehi-miR-24, Ehi-miR-29, Ehi-miR-46, Ehi-miR-47, Ehi-miR-144) and used rRNA 18S as control. The results shown in Figure S3 suggest that 9 out of 10 RT-PCR runs showed a level of amplification validating the presence of these candidate miRNAs.

## Discussion

In cells, precise regulation of gene and protein expression is a fundamental mechanism for development, homeostasis, and adaptation to the environment. In eukaryotes, every step in the process of gene expression is subject to dynamic regulation. Studies have identified diverse biological processes involved in the regulation of gene expression in protozoa parasites.

*Entamoeba histolytica* is a parasite that causes amebic dysentery and liver abscesses. The parasite and host factors that control the outcome of this infection are not well understood, although there is emerging evidence of host, parasite, and environmental factors influencing the outcome of infection [1], [2], [3]. Altered transcription of certain crucial genes is also likely to contribute to the outcome of infection. The latent period between infection and disease in humans suggests that the parasite adapts to the host via altered gene expression [4]. Over the past decade, it has become clearer that a large class of small noncoding RNAs, known as microRNAs (miRNAs), function as important regulators of a wide range of cellular processes by modulating the translation and degradation of mRNAs. However, miRNA mechanisms have not been studied in *Entamoeba histolytica*. To continue the rapid advancements surrounding miRNA discoveries we need applicable and validated experimental tools to enable researchers to study the expression and biological function of miRNAs.

Next-generation sequencing became available for the sequencing of small RNA molecules, including miRNAs. The sensitivity of deep sequencing allows us to measure absolute abundance and aids in the discovery of novel miRNAs. In this study, we obtained 16 million raw reads from a purified fraction of about 22 nt containing a total 5,239,324 mappable sequences with high-quality reads.

We found that only 0.5% of these non-redundant small RNA reads were a perfect match with the draft *E. histolytica* genome and were candidates to be miRNAs because their corresponding genomic region showed potential to form hairpins.

We experimentally identified the first 199 putative *E. histolytica* miRNAs according to criteria for distinguishing bilaterian miRNAs from other types of small RNAs [10], [19], [20] currently implemented in the bioinformatics pipeline script ACGT101-miR v4.2 [21], [22]. Surprisingly, we found no similarities with known plant and animal miRNAs. This result is consistent with the finding that *Chlamydomonas reinhardtii,* a unicellular green alga, contains no homolog miRNAs. [23] This result suggests that unicellular miRNAs may represent a novel class of miRNAs that deserves to be studied further. Additionally, the lack of universally conserved miRNA among plants, animals, and green algae suggests that miRNA genes may have evolved independently in the lineages leading to these groups [23]. Our results suggest that the evolution of *E. histolytica* could be similar to that of green algae.

De and his colleagues identified seventeen candidate microRNAs in *Entamoeba histolytica* using a bioinformatic approach [9]; we have not observed similarities between the 199 miRNAs obtained in this study and those proposed by De et al. This difference may be due to these three factors: the different genome assemblies–De et al. used data from The Institute of Genome Research (TIGR) *Entamoeba* genome database [24] while we used data from AmoebaDB (EuPathDBs) [25]; to the lack of representativity of the transcripts in the sample used in our study (trophozoite) or; to unknown deficiencies in the bioinformatics pipelines used in both studies.

We confirmed by microarray the presence of all predicted miRNA candidates. Additionally, Real-time PCR has proven to be a simple and accurate method to identify and measure the expression levels of miRNAs [26]. Using this approach, we validated the presence of 9 out of 10 selected novel miRNAs.

As an initial step toward understanding the biological function of miRNAs in *E. histolytica*, we searched for miRNA targets among annotated protein-coding transcripts of *E. histolytica*. Applying a cutoff value of −18 kCal mol^−1^, we predicted a total of 32 genes as miRNA targets (Table S2). The putative target genes appear to be involved in various biological processes. However, since the *E. histolytica* genome is not fully annotated and a great proportion of *E. histolytica* protein-coding genes have no known function, it is difficult to draw a conclusion as to whether these miRNA targets have any functional bias.

Our results suggest that miRNA-controlled mechanisms may be involved in human infection of *E. histolytica.* Some of the predicted target genes with annotated functions that we found are involved in gene regulation (zinc finger protein) and signal transduction, such as Ras family GTPase. These proteins have been implicated in key pathogenic processes of *E. histolytica*. Some Ras and Rho GTPase effectors, particularly kinases such as the PAKs and members of the mitogen-activated protein kinase cascade, have also proven tractable as pharmacological targets in humans [27], [28]. However, the importance of Ras effectors and downstream kinases in *E. histolytica* pathogenesis has not yet been explored [29].

The liver is also a prime target for amoeba infection. This organ contains a plentiful source of iron, which is essential for the growth of this parasite. Amoeba trophozoites are able to take up ferritin from the liver and internalize this protein via clathrin-coated vesicles. The capacity to use ferritin as an iron source may explain *E. histolytica’*s high pathogenic potential in the liver [30]. Since clathrin is a protein required for receptor-mediated internalization of lipid and protein molecules, [31], [32] its regulation by miRNAs as potential target detected here could help to reduce the pathogenic functions of this parasite in the liver.

Another predicted target, the TolA protein, is involved in the translocation of group A colicins. The colicins are bacterial proteins that are active against *Escherichia coli* and other related species. TolA is anchored to the cytoplasmic membrane by a single membrane-spanning segment near N-terminus, leaving most of the protein exposed to the periplasm [33].

It is noteworthy that one miRNA regulates the unconventional myosin IB protein. The pathogenicity of *E. histolytica* includes its capacity to phagocyte human cells. Motility requires polarization of *E. histolytica* that involves protrusion of a pseudopod containing actin and associated proteins (myosin IB, ABP-120 and a p21-activated kinase [PAK]) and whole-cell propulsion after contraction of the rear of the cell, where myosin II and F-actin are concentrated. An interesting characteristic of *E. histolytica* is the presence of two unique myosins (myosin II and unconventional myosin IB) in contrast to several actin genes. as been proposed that survival and pathogenicity of *E. histolytica* require an active actin-myosin cytoskeleton to cap surface receptors, adhere to host components, migrate through tissues, and phagocyte human cells and liver abscesses [34].

Finally, the recent discovery that miRNAs are present in blood, plasma, serum, and other fluids like urine and saliva, has raised the interest of their use as potential biomarkers and diagnostic tools [35]. The presence of miRNA molecules in those biological fluids is attributed both to their stability and small size. It has also been demonstrated that the majority of miRNAs detectable in serum and saliva are found inside exosomes that could avoid miRNA degradation and serve as transport particles to facilitate miRNA actions in neighboring cells [36].

The presence and relative concentration of specific miRNAs in different biological fluids is related with the tissue, and also with the physiological status of the tissue, resulting in the expression of defined protein expression profiles, as demonstrated for several pathologies [36], [37]. This difference could be exploited for the specific diagnosis of defined infectious agents, by using novel technologies that allow the detection of sub-picomolar levels of miRNAs in biological fluids like plasma samples, since these technologies could discriminate single nucleotide differences between miRNA family members [11].

The combination between the characteristics of the miRNAs (presence in biological fluids and thus ease of collection, stability in those samples), and the possibility of differentiating various organisms through their specific miRNA sequences, should raise the interest in the detection of miRNA as diagnostic tools for parasitic diseases, an utility that has been already shown for other diseases [38].

Taking in account all these considerations, detection of microRNAs in *E. histolytica* described in this paper could be used as potential biomarkers in the specific diagnosis of amoebiasis using biological fluids.

In conclusion we identified 199 potential miRNAs by deep sequencing of short RNAs from *Entamoeba histolytica* trophozoites. This study represents the first characterization of miRNA transcriptome in this parasite and could be used as a new platform to study the genomic structure, gene regulation and evolutionary processes of *E. histolytica* as well as host-parasite interactions.

## Materials and Methods

### Culture of Trophozoites of *Entamoeba histolytica* Strain HM1-IMSS

Trophozoites in log phase of *E. histolytica* strain HM1-IMSS were cultivated (2×10^4^ cells/ml) in 18×150 mm borosilicate screw-capped tubes containing 20 mL of TYI-S-33 medium [39], 4.0 mL bovine serum (bovine serum sterilized by gamma radiation at 2 mrads), and 0.2 mL antibiotic mixture (50,000 IU penicillin/ml and streptomycin 50 mg/ml) and incubated at 37°C for four days. The tubes were cooled at 4°C for 20 minutes and then centrifuged at 978 *g* for 10 min at room temperature, the TYI-S-33 medium was eliminated and the pellet was washed three times with PBS pH 7.4.

### RNA Extraction

We isolated total RNA that contained small RNA from 25 mg of cells using miRNAeasy kit (Qiagen, Valencia, CA) according to the manufacturer’s protocol. RNA was eluted with 50 µL of RNAse free water. We quantified the concentration of all RNA samples using NanoDrop 1000 (Thermo Scientific, Wilmington, DE).

### Sequencing and Analysis of E. Histolytica miRNAs

miRNAs were sequenced by LC Sciences (Houston, TX). In brief, small RNA fraction of 15–50 nt from trophozoites of *E. histolytica* total RNA was isolated from a 15% Tris-Borate-EDTA-Urea polyacrylamide gel. Following ligations of the small RNAs with adaptors (Illumina, San Diego, CA), the 64–99-nt-long RNAs were isolated through gel elution and ethanol precipitation. A small RNA library was generated using the Illumina TruseqTM Small RNA Preparation kit according to the manufacturer’s guidelines. The purified cDNA library was used for cluster generation on Illumina’s Cluster Station and then sequenced on Illumina GAIIx following vendor’s instructions. Raw sequencing reads (40 nt) were obtained using Illumina’s Sequencing Control Studio software version 2.8 (SCS v2.8) following real-time sequencing image analysis and base-calling by Illumina’s Real-Time Analysis version 1.8.70 (RTA v1.8.70). Sequencing data analysis was performed by proprietary pipeline script, ACGT101-miR v4.2 (LC Sciences) [21], [22], [40].

### Identification of Putative miRNAs

We used the following criteria to identify potential miRNAs: (1) reads mapped to an inferred RNA hairpin with pairing characteristics of known miRNA hairpins; (2) the seed sequence is similar to known miRNAs from other species (miRBase release 18.0) [12]; and (3) the RNA was not mapped to a genomic region with an annotation suggesting a non-miRNA biogenesis [19], [20], [41]. For this, we used a proprietary pipeline script, ACGT101-miR v4.2 (LC Sciences, Houston, TX, USA), to predict novel miRNAs [21], [22]. The complete genome of *Entamoeba histolytica* (http://amoebadb.org) was used as reference for annotation of *Entamoeba histolytica* miRNAs. All clean reads were mapped using several databases including GenBank, Rfam, animal miRNAs (miRBase) and Repbase.

### Paraflo™ MicroRNA Microarray Assay

Microarray assay was performed using LC Sciences. The assay started from a 4-to-8-µg total RNA sample whose 3′ was extended with a poly(A) tail using poly(A) polymerase. An oligonucleotide tag was then ligated to the poly(A) tail for later fluorescent dyeing. Hybridization was performed overnight on a µParaflo microfluidic chip using a micro-circulation pump (Atactic Technologies) [42]. On the microfluidic chip, each detection probe consisted of a chemically modified nucleotide coding segment complementary to target microRNA (from miRBase, http://mirbase.org) or other RNA (control or customer defined sequences) and a spacer segment of polyethylene glycol to extend the coding segment away from the substrate. The detection probes were made by *in situ* synthesis using PGR (photogenerated reagent) chemistry. The hybridization melting temperatures were balanced by chemical modifications of the detection probes. Hybridization used 100 µL 6xSSPE buffer (0.90 M NaCl, 60 mM Na_2_HPO_4_, 6 mM EDTA, pH 6.8) containing 25% formamide at 34°C. After RNA hybridization, tag-conjugating Cy3 dye was circulated through the microfluidic chip for staining. Fluorescence images were collected using a laser scanner (GenePix 4000B, Molecular Device) and digitized using Array-Pro image analysis software (Media Cybernetics). Data were analyzed by first subtracting the background and then normalizing the signals using a LOWESS filter (Locally-weighted Regression) [43].

### Real Time PCR

We used SYBR green PCR assay. In brief, 500 ng of *Entamoeba histolytica* RNA was polyadenylated and reverse transcribed to cDNA using the High-Specificity miRNA QPCR Core reagent kit from Agilent. Real-time PCR was performed on the Stratagene Mx3005P real-time PCR system. The miRNA-specific forward primer sequences were designed based on the miRNA sequences obtained from this work. The miRNA-specific primer sequences for Real Time PCR are listed in Table S3. Each sample was run in duplicates for analysis.

## Supporting Information

Figure S1 **Data analysis flowchart.** Sequencing data analysis was performed by proprietary pipeline script, ACGT101-miR v4.2 from LC Sciences.
(TIF)

Figure S2 **Pie plot of database mapping.** Number of mappable reads for each group.
(TIF)

Figure S3 **Amplification plot of Real Time PCR.** The results suggest that 9 out of 10 RT-PCR runs showed a level of amplification validating the presence of these candidate miRNAs.
(TIF)

Table S1 (XLSX)

Table S2 (DOC)

Table S3 (DOC)
