# Supplementary figures and images for: Identification and Characterization of microRNAS from Entamoeba histolytica HM1-IMSS
Source: PLoS One. 2013 Jul 12;8(7):e68202. doi: 10.1371/journal.pone.0068202 (PMC3709888; doi:10.1371/journal.pone.0068202)

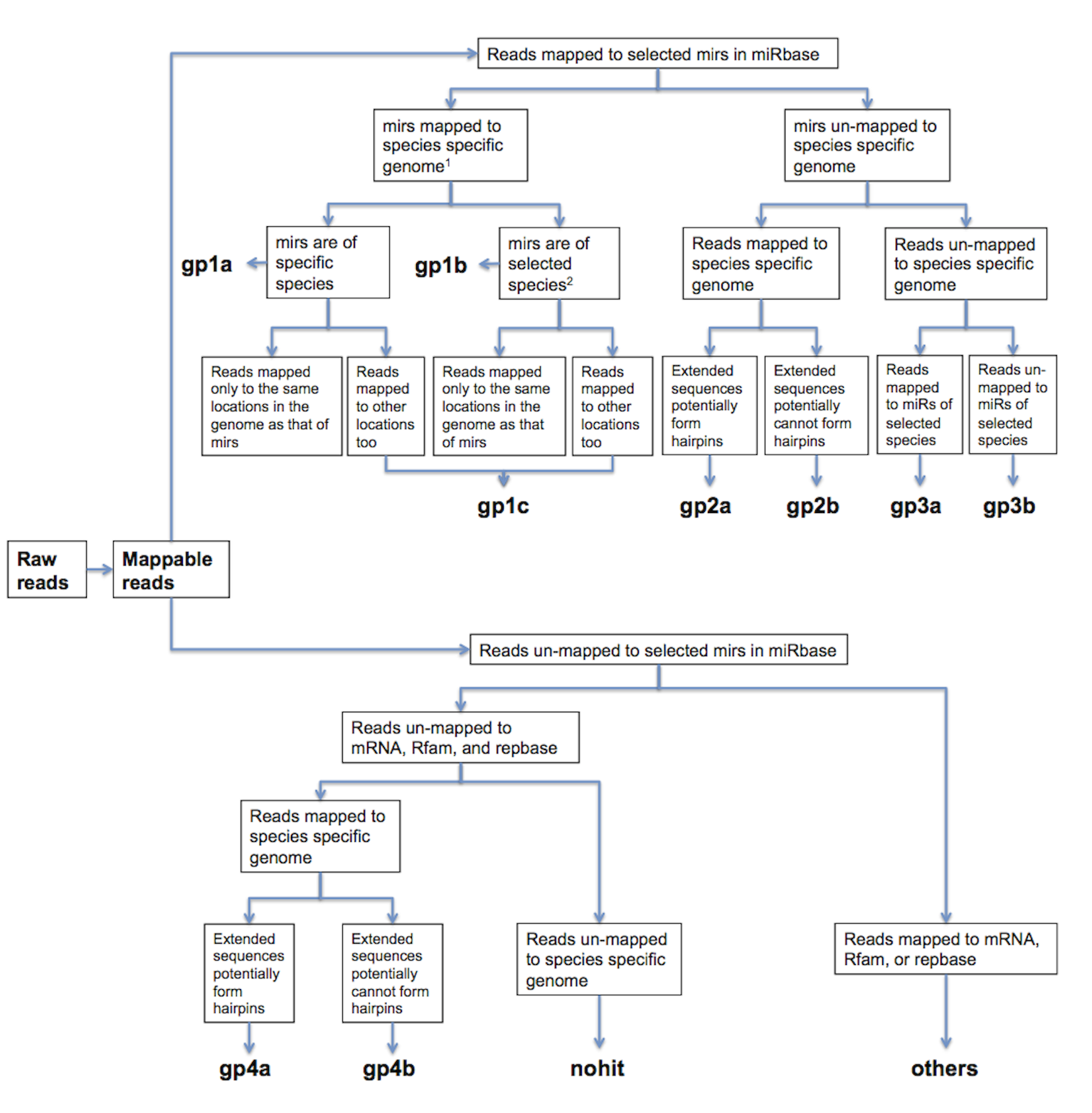

Supplement: Figure S1 — Data analysis flowchart. Sequencing data analysis was performed by proprietary pipeline script, ACGT101-miR v4.2 from LC Sciences. (TIF) [file pone.0068202.s001.tif]

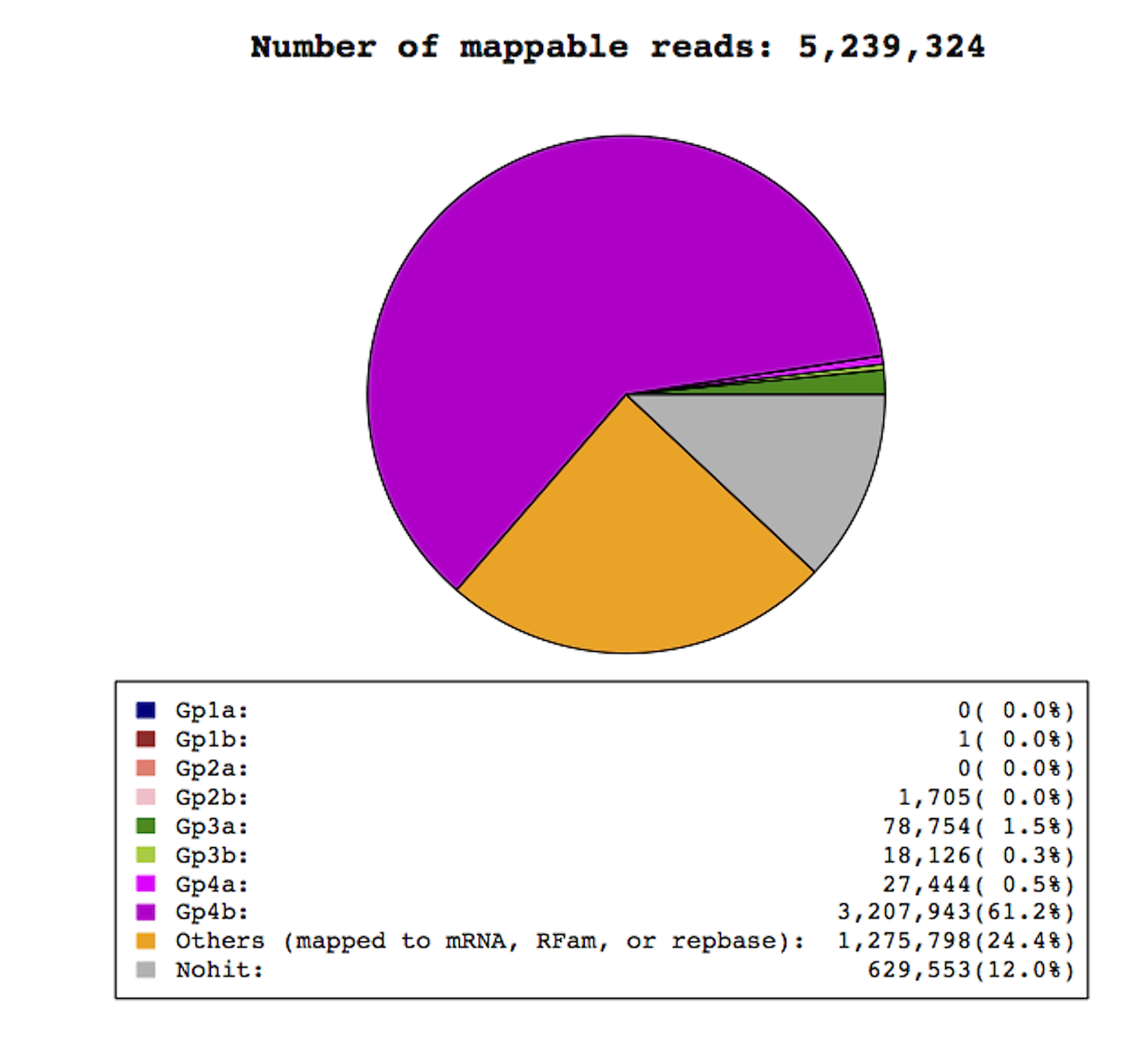

Supplement: Figure S2 — Pie plot of database mapping. Number of mappable reads for each group. (TIF) [file pone.0068202.s002.tif]

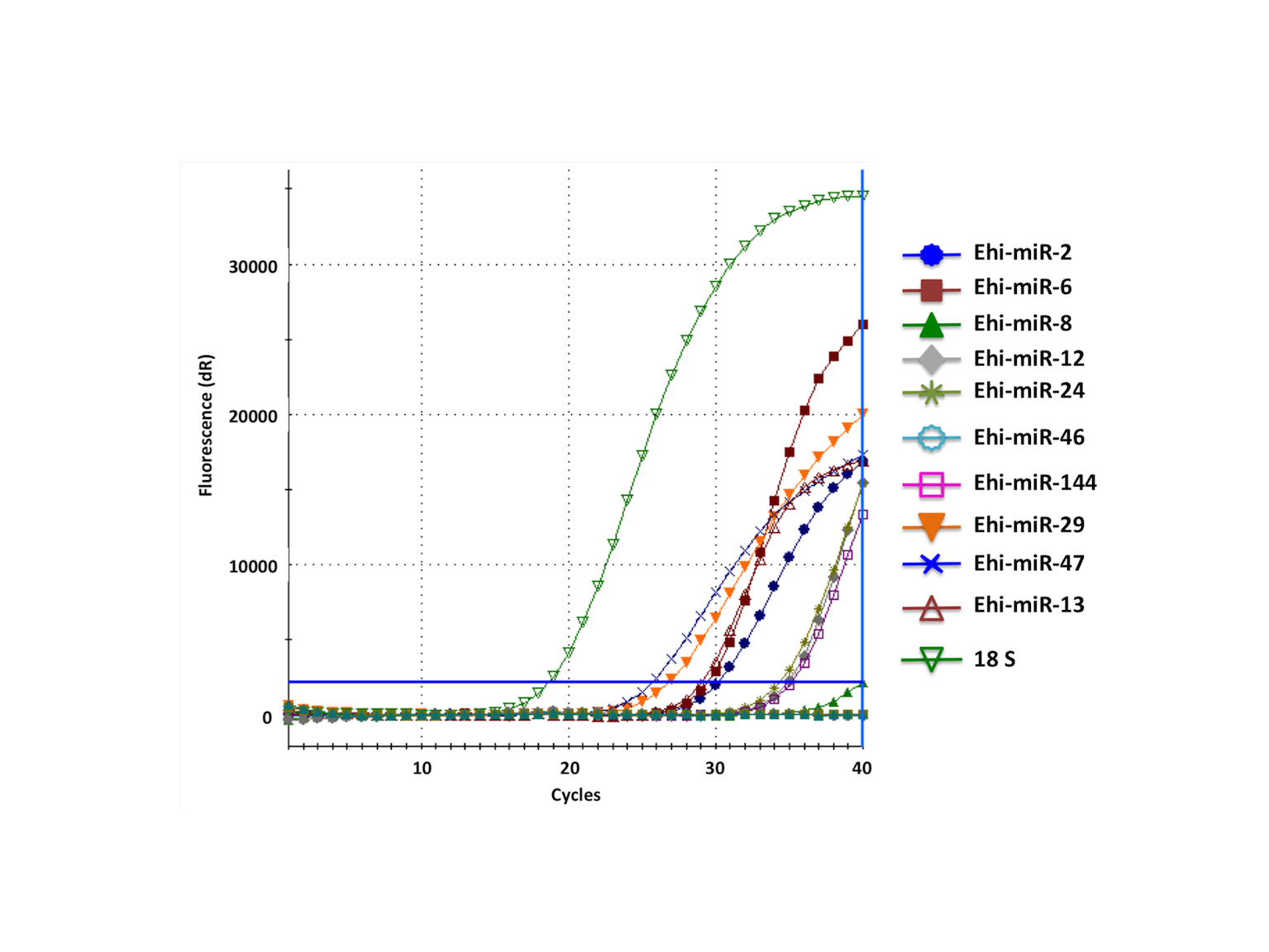

Supplement: Figure S3 — Amplification plot of Real Time PCR. The results suggest that 9 out of 10 RT-PCR runs showed a level of amplification validating the presence of these candidate miRNAs. (TIF) [file pone.0068202.s003.tif]
