# Supplementary material for: Identification and Characterization of microRNAS from Entamoeba histolytica HM1-IMSS
Source: PLoS One. 2013 Jul 12;8(7):e68202. doi: 10.1371/journal.pone.0068202 (PMC3709888; doi:10.1371/journal.pone.0068202)
Supplement: Table S2 — (DOC) [file pone.0068202.s005.doc]

|  | Gene | Description |
| --- | --- | --- |
| 1 | EHI_107290 | EHI_107290 | Entamoeba histolytica HM-1:IMSS | actin, putative | genomic | |
| 2 | EHI_110810 | Entamoeba histolytica HM-1:IMSS | unconventional myosin IB | genomic | |
| 3 | EHI_069320 | EHI_069320 | Entamoeba histolytica HM-1:IMSS | C2 domain containing protein | genomic | |
| 4 | EHI_038330 | EHI_038330 | Entamoeba histolytica HM-1:IMSS | zinc finger domain containing protein | |
| 5 | EHI_050900 | Entamoeba histolytica HM-1:IMSS | N-system amino acid transporter 1, putative | genomic | |
| 6 | EHI_124610 | Entamoeba histolytica HM-1:IMSS | Ras family GTPase | genomic | |
| 7 | EHI_159620 | Entamoeba histolytica HM-1:IMSS | TolA protein, putative | genomic | |
| 8 | EHI_186830 | Entamoeba histolytica HM-1:IMSS | 60S acidic ribosomal protein P2, putative | genomic | |
| 9 | EHI_194540 | Entamoeba histolytica HM-1:IMSS | pore-forming peptide ameobapore B precursor, putative | genomic | |
| 10 | EHI_048680 | Entamoeba histolytica HM-1:IMSS | 60S ribosomal protein L12, putative | genomic | |
| 11 | EHI_114390 | Entamoeba histolytica HM-1:IMSS | ABC transporter, putative | genomic | |
| 12 | EHI_009940 | Entamoeba histolytica HM-1:IMSS | clathrin adaptor complex small chain, putative | genomic | |
| 13 | EHI_017700 | Entamoeba histolytica HM-1:IMSS | ribosomal protein L13, putative | genomic | |
| 14 | EHI_023600 | Entamoeba histolytica HM-1:IMSS | adaptor protein (AP) family protein | genomic | |
| 15 | EHI_030710 | Entamoeba histolytica HM-1:IMSS | 60S ribosomal protein L12, putative | genomic | |
| 16 | EHI_038860 | Entamoeba histolytica HM-1:IMSS | 40S ribosomal protein S28, putative | genomic | |
| 17 | EHI_053840 | Entamoeba histolytica HM-1:IMSS | thioredoxin, putative | genomic | |
| 18 | EHI_072040 | Entamoeba histolytica HM-1:IMSS | 40S ribosomal protein S3a, putative | genomic | |
| 19 | EHI_088490 | Entamoeba histolytica HM-1:IMSS | protein kinase, putative | genomic | |
| 20 | EHI_091080 | Entamoeba histolytica HM-1:IMSS | TBC domain containing protein | genomic | |
| 21 | EHI_118640 | Entamoeba histolytica HM-1:IMSS | CBF/NF-Y transcription factor domain protein | genomic | |
| 22 | EHI_127360 | Entamoeba histolytica HM-1:IMSS | anti-silencing protein, putative | genomic | |
| 23 | EHI_137700 | Entamoeba histolytica HM-1:IMSS | Ras family GTPase | genomic | |
| 24 | EHI_138500 | Entamoeba histolytica HM-1:IMSS | Rap/Ran GTPase-activating protein, putative | genomic | |
| 25 | EHI_140550 | Entamoeba histolytica HM-1:IMSS | 60S ribosomal protein L6, putative | |
| 26 | EHI_146340 | Entamoeba histolytica HM-1:IMSS | 40S ribosomal protein S3, putative | genomic | |
| 27 | EHI_151920 | Entamoeba histolytica HM-1:IMSS | copine inorganic polyphosphate/ATP-NAD kinase, putative | genomic | |
| 28 | EHI_152570 | Entamoeba histolytica HM-1:IMSS | 60S ribosomal protein L26, putative | genomic | |
| 29 | EHI_163260 | Entamoeba histolytica HM-1:IMSS |, putative | genomic | |
| 30 | EHI_178890 | Entamoeba histolytica HM-1:IMSS | eukaryotic translation initiation factor 3 subunit 1, putative | genomic | |
| 31 | EHI_182460 | Entamoeba histolytica HM-1:IMSS | dextranase precursor, putative | genomic | |
| 32 | EHI_187770 | Entamoeba histolytica HM-1:IMSS | SH3 domain protein | genomic | |
| 33 | EHI_194520 | Entamoeba histolytica HM-1:IMSS | LIM zinc finger domain containing protein | genomic | |
| 34 | EHI_200780 | Entamoeba histolytica HM-1:IMSS | TBC domain containing protein | genomic | |
