# Supplementary material for: Identification and Characterization of microRNAS from Entamoeba histolytica HM1-IMSS
Source: PLoS One. 2013 Jul 12;8(7):e68202. doi: 10.1371/journal.pone.0068202 (PMC3709888; doi:10.1371/journal.pone.0068202)
Supplement: Table S3 — (DOC) [file pone.0068202.s006.doc]

|  | **miRNA** | **Sequence** | **Tm** |
| --- | --- | --- | --- |
| **1** | **Ehi-miR-2** | **GGTCTAGGATTCTGTCT** | **54°C** |
| **2** | **Ehi-miR-5** | **CATGATCTGAAGGGATGA** | **55°C** |
| **3** | **Ehi-miR-8** | **GAATAAATTGTAATACTCGATT** | **50°C** |
| **4** | **Ehi-miR-12** | **AATAAACGAAGGAACATCTATTT** | **55°C** |
| **5** | **Ehi-miR-24** | **AGTTCAATGAAGCACG** | **54°C** |
| **6** | **Ehi-miR-46** | **ATACCTCCTGAACCAAATA** | **54°C** |
| **7** | **Ehi-miR-144** | **CTTAGTGGTGATAGATCG** | **53°C** |
| **8** | **Ehi-miR-29** | **TAATTAAGGATAGTAAGTGG** | **50°C** |
| **9** | **Ehi-miR-47** | **TCATTTACACCTTATTTATTAGG** | **53°C** |
| **10** | **Ehi-miR-13** | **CTCGTCTATTATTTTCTTCAAATC** | **55°C** |
| **11** | **Eh-18S5’** | **CAGTTTCATCCTACTTGGTC** | **58°C** |
| **12** | **Eh-18S3’** | **ATCCTGCCAGTATTATATGC** | **57°C** |
